# Molecular characterization of a mutation affecting abscisic acid biosynthesis and consequently stomatal responses to humidity in an agriculturally important species

**DOI:** 10.1093/aobpla/plv091

**Published:** 2015-07-27

**Authors:** Scott A. M. McAdam, Frances C. Sussmilch, Timothy J. Brodribb, John J. Ross

**Affiliations:** School of Biological Sciences, University of Tasmania, Private Bag 55, Hobart, TAS 7005, Australia

**Keywords:** Abscisic acid (ABA), biosynthesis, evolution, humidity, *Pisum sativum*, stomata, vapour pressure deficit, *wilty* mutant

## Abstract

Mutants deficient in the phytohormone abscisic acid (ABA) have been instrumental in determining not only the biosynthetic pathway for this hormone, but also its physiological role in land plants. The *wilty* mutant of *Pisum sativum* is one of the classical, well-studied ABA-deficient mutants; however, this mutant remains uncharacterized at a molecular level. Using a candidate gene approach, we show that the *wilty* mutation affects the xanthoxin dehydrogenase step in ABA biosynthesis. To date, this step has only been represented by mutants in the *ABA2* gene of *Arabidopsis thaliana*. Functional ABA biosynthesis appears to be critical for normal stomatal responses to changes in humidity in angiosperms, with *wilty* mutant plants having no increase in foliar ABA levels in response to a doubling in vapour pressure deficit, and no closure of stomata. Phylogenetic analysis of the *ABA2* gene family from diverse land plants indicates that an ABA-biosynthesis-specific short-chain dehydrogenase (ABA2) evolved in the earliest angiosperms. The relatively recent origin of specificity in this step has important implications for both the evolution of ABA biosynthesis and action in land plants.

## Abstract (precis)

Mutants deficient in the phytohormone abscisic acid (ABA) have been instrumental physiological models for understanding both the biosynthesis and action of this critical hormone. The *wilty* mutant of the agriculturally important species *Pisum sativum* is a quintessential ABA deficient mutant which has remained molecularly uncharacterised in the 40 years since its discovery. We show that the *wilty* mutation affects the xanthoxin dehydrogenase step in the ABA biosynthetic pathway and that this step in ABA biosynthesis is critical for normal stomatal responses to changes in humidity.

## Introduction

**Figure 1.**
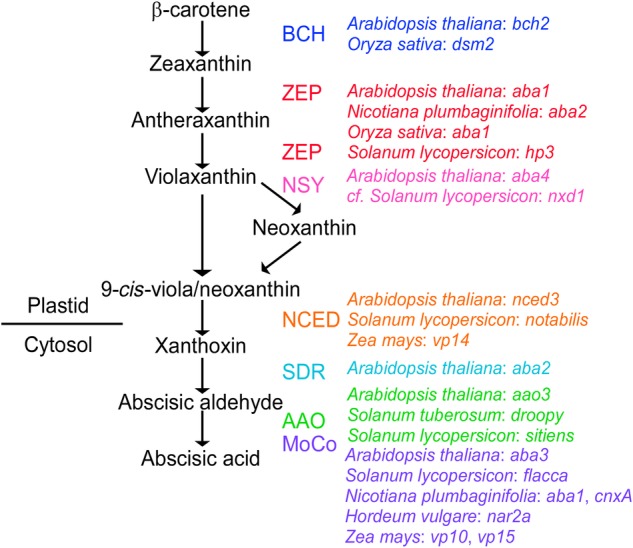
The biosynthetic pathway for ABA from the carotenoid, β-carotene; known enzymes and characterized mutants of these enzymes at each step are indicated.

The phytohormone abscisic acid (ABA) is critical for land plant survival and is implicated in plant responses to water deficit. One of the earliest identified roles for ABA is closing the stomata of angiosperms (Mittelheuser and Van Steveninck 1969; Jones and Mansfield 1970). The signalling pathway for ABA-induced stomatal closure has been the subject of detailed investigation in recent years, through the use of single gene mutants, and is reasonably well understood (Geiger *et al*. 2011). Likewise, mutants deficient in ABA levels have been instrumental in revealing the pathway by which ABA is synthesized in plants (Nambara and Marion-Poll 2005; Taylor *et al*. 2005). The molecular characterization of mutants from diverse angiosperm species has provided strong evidence for the widely accepted, linear biosynthetic pathway for this hormone (Fig. 1).

In land plants, ABA is synthesized from the precursor β-carotene, a carotenoid (Fig. 1). β-Carotene biosynthesis mutants have pleiotropic phenotypes, but mutations affecting later steps of ABA biosynthesis result specifically in ABA deficiency (Taylor *et al*. 2000). After the formation of β-carotene, the next step involves the hydroxylation of β-carotene to zeaxanthin, a critical xanthophyll (Fig. 1). Mutants defective in β-carotene hydroxylase (*BCH*) in *Arabidopsis thaliana* (*bch2*) and *Oryza sativa* (*dsm2*) have weak control of transpiration and low levels of ABA (Kim *et al*. 2009; Du *et al*. 2010; Águila Ruiz-Sola and Rodríguez-Concepción 2012). Zeaxanthin epoxidase (ZEP) then converts zeaxanthin to violaxanthin via the xanthophyll cycle (Fig. 1). Pronounced ABA deficiency is well described in mutants of *ZEP* from a range of species including *A. thaliana* (*aba1*) (Koornneef *et al*. 1982; Barrero *et al*. 2005), *Nicotiana plumbaginifolia* (*aba2*) (Marin *et al*. 1996), *Solanum lycopersicon* (*hp3*) (Galpaz *et al*. 2008) and *O. sativa* (*aba1*) (Agrawal *et al*. 2001). Violaxanthin is then converted to neoxanthin by neoxanthin synthase (Fig. 1); however, this step is known from only a single ABA-deficient mutant, in *A. thaliana* (*aba4*) (North *et al*. 2007) and is redundant in *S. lycopersicon* (Neuman *et al*. 2014). Isomerization of neoxanthin and/or violaxanthin from 9-*trans* to 9-*cis* then occurs by an unknown mechanism (Fig. 1).

Oxidative cleavage of 9-*cis*-neoxanthin and/or 9-*cis*-violaxanthin to xanthoxin is catalyzed by a 9-*cis*-epoxycarotenoid dioxygenase (NCED), in the chloroplasts (Tan *et al*. 2001, 2003). This is the rate-limiting step in ABA biosynthesis, and the first non-reversible step (Qin and Zeevaart 1999; Thompson *et al*. 2000). Of note, *NCED* mutants have been identified in *Zea mays* (*vp14*) (Schwartz *et al*. 1997), *A. thaliana* (*nced3*) (Iuchi *et al*. 2001; Ruggiero *et al*. 2004; Huang *et al*. 2008) and *S. lycopersicon* (*notabilis*) (Burbidge *et al*. 1999) and all have severe deficits in ABA, particularly under water stress. Once formed, xanthoxin moves across the membrane of the chloroplast to the cytosol and is converted to abscisic aldehyde by a short-chain alcohol dehydrogenase/reductase (SDR) (Fig. 1). Thus far, *A. thaliana aba2* is the only mutant identified that affects this step (Cheng *et al*. 2002; González-Guzmán *et al*. 2002). However, an *O. sativa* ortholog of *ABA2* has been found to restore the ABA-deficient phenotype in *A. thaliana aba2* mutants, suggesting that the function of this gene may be conserved across angiosperms (Endo *et al*. 2014). The final step in the ABA-biosynthetic pathway is the conversion of abscisic aldehyde to ABA by an abscisic aldehyde oxidase (*AAO*) gene (Fig. 1). Mutants of this final step are well represented in diverse species, including *S. lycopersicon* (*sitiens*) (Harrison *et al*. 2011), *S. tuberosum* (*droopy*) [based on synteny between the genomes of the two *Solanum* species (Duckham *et al*. 1989)] and *A. thaliana* (*aao3*) (Seo *et al*. 2000). The activity of AAO requires a molybdenum cofactor (MoCo) and mutants defective in the synthesis or maturation of this MoCo suffer from ABA deficiency in *S. lycopersicon* (*flacca*) (Sagi *et al*. 2002), *Hordeum vulgare* (*nar2a*) (Walker-Simmons *et al*. 1989), *N. plumbaginifolia* (*aba1* and *cnxA*) (Leydecker *et al*. 1995; Mendel and Schwarz 1999), *A. thaliana* (*aba3* and other alleles) (Bittner *et al*. 2001; Xiong *et al*. 2001) and *Z. mays* (*vp10* and *vp15*) (Porch *et al*. 2006; Suzuki *et al*. 2006).

This collection of classical ABA-deficient mutants has also been critical for informing our understanding of the roles ABA plays in angiosperm physiology and survival, including driving stomatal closure, inducing seed dormancy and aiding desiccation and salinity tolerance. A quintessential ABA-deficient mutant used in physiological studies on *Pisum sativum* is *wilty. wilty* was identified from a spontaneous mutation in an unknown breeding line nearly 40 years ago (Marx 1976), but has not been previously characterized at a molecular level. The *wilty* mutant has provided insights into the physiological role of ABA in seed development (de Bruijn and Vreugdenhil 1992; de Bruijn *et al*. 1993*b*; Batge *et al*. 1999), plant growth (de Bruijn *et al*. 1993*a*) and stomatal behaviour (Donkin *et al*. 1983). *wilty* plants display a loss of turgor under conditions of moderate evaporative demand, and have particularly low ABA levels in seeds (10-fold less than wild-type plants) (Batge *et al*. 1999) and leaves, especially during drought-stress (Wang *et al*. 1984). Rapid reductions in the water status of the leaves of *wilty* compared with wild-type plants, during desiccation, have been well documented (Donkin *et al*. 1983; de Bruijn *et al*. 1993*a*). A single study has attempted to identify the biosynthetic step of ABA that is impaired in *wilty* plants, finding that *wilty* plants were able to rapidly synthesize ABA after the application of radiolabelled abscisic aldehyde (Duckham *et al*. 1989). This has led to the suggestion that the gene impaired in the *wilty* mutant is upstream of this step in the biosynthetic pathway and possibly due to a lesion in the *NCED* gene responsible for the conversion of 9*-cis*-neoxanthin or 9-*cis*-violaxanthin to xanthoxin (Taylor *et al*. 2000). However, this has never been further examined.

In this study, we provide evidence regarding the molecular basis of the *wilty* mutation of *P. sativum*. The evidence strongly suggests that a mutation in the *P. sativum* ortholog of *ABA2* underlies the *P. sativum wilty* mutant phenotype. We use this mutant to investigate the role of ABA biosynthesis in the rapid stomatal response to changes in humidity or vapour pressure deficit (VPD). There are mixed reports in the literature relating to the stomatal responses to VPD in ABA-biosynthetic mutants, although all work to date has focussed on *A. thaliana* and none have measured ABA levels (Assmann *et al*. 2000; Xie *et al*. 2006; Bauer *et al*. 2013; Merilo *et al*. 2015)*.* Conclusions have ranged from ABA-biosynthetic mutants in *A. thaliana* having normal stomatal response to changes in VPD (Assmann *et al*. 2000) to ABA biosynthesis being critical for stomatal responses to such changes (Bauer *et al*. 2013). Recently, it has been shown that ABA levels in angiosperms can increase over a very short period (20 min) in response to a reduction in relative humidity, and that this increase in foliar ABA level is responsible for the closing of stomata, particularly in angiosperm herbs (McAdam and Brodribb 2015). Here we investigate whether plants that have an inability to synthesize ABA have functional stomatal response to changes in humidity.

## Methods

### Plant material

The original *wilty* line resulted from a spontaneous mutation that emerged in a breeding line belonging to L.G. Cruger from Del Monte Corporation, San Lenadro, CA, and was later sent to Prof. G.A. Marx in the early 1970s (Marx 1976). This original line, which has no known wild type, has been used in all physiological studies using *wilty* to date and is referred to in this study as L233. All physiological experiments here use line *wil* which was derived by introgression of L233 with the cultivar Torsdag over five backcrosses to create a near isogenic line. Eight lines of *P. sativum* were used to represent wild-type genotypes for sequencing of *ABA2* in this study. These included JI281, WL1771, the *argenteum* mutant [also a spontaneous mutation that emerged in a breeding line belonging to L.G. Cruger, with an unknown background (Marx 1982)] and the cultivars Cameor, Champagne, Kaliski, Torsdag (Hobart Line 107) and Virtus. In addition to these diverse *P. sativum* lines, a single representative from the closely related genus *Lathyrus odoratus* [Hobart Line LO5, derived from the cultivar Grandiflora XD by Ross and Murfet (1985)] was also used.

### Stomatal responses to changes in VPD

Plants were grown in a growth cabinet (PGC-105, Percival Scientific Inc.) in 2.6 L 14 × 17 cm slim-line pots in a 1 : 1 mix of vermiculite and dolerite gravel chips topped with 3 cm of an 8 : 1 mix of composted pine bark and course river sand. All plants were watered daily and received weekly applications of liquid fertilizer (Aquasol, Hortico Ltd). Conditions in the growth cabinet were regulated at 25 °C/16 °C day/night temperature and a 16 h photoperiod provided by mixed incandescent and fluorescent lights ensuring a minimum 300 μmol quanta m^−2^ s^−1^ at the pot surface. Before the experiment, a daytime VPD of 1.2 kPa (62 % relative humidity) was maintained while temperature and relative humidity were monitored every 5 min during this period by a data logger (HOBO Pro Series, Onset). Plants were initially grown at a VPD of 1.2 kPa to allow leaves to expand under relatively low humidity so as to avoid the potential stomatal dysfunction apparent in leaves that have expanded under very high humidity (Aliniaeifard *et al*. 2014).

When the plants were 3 weeks old, with approximately seven fully expanded leaves, the VPD in the cabinet was lowered to 0.7 kPa (±0.05 kPa) by the presence of containers of water and a 1 m^2^ surface of wet hessian. After a 5-day acclimation period to this low VPD the simultaneous monitoring of leaf gas exchange and ABA levels were undertaken on leaves from seven individuals of *wil* and Torsdag (as described below). After this initial simultaneous measurement of leaf gas exchange and ABA level, VPD was increased to 1.5 kPa (42 % relative humidity) using a condensing dehumidifier (SeccoUltra 00563, Olimpia-Splendid) in the growth cabinet. Temperature and relative humidity were monitored every 30 s during the experimental period by a humidity probe (HMP45AC, Vaisala) and thermocouple connected to a data logger (CR10X, Campbell Scientific). A VPD of 1.5 kPa was maintained for 20 min after which leaf gas exchange and ABA level were again simultaneously measured in leaves from three individuals. Vapour pressure deficit was then returned to 0.7 kPa and measurements were again conducted after a further 20 min. The relatively small and contained volume of air in the growth cabinet (3 m^3^) resulted in a relatively fast half time for the VPD transition of 150 s following transitions between 0.7 and 1.5 kPa (with no hysteresis when VPD was returned to 0.7 kPa).

### Leaf gas exchange measurements

During transitions in VPD, leaf gas exchange was measured in fully irradiated (300 µmol quanta m^−2^ s^−1^) leaves using an infrared gas analyser (LI-6400, LI-COR Biosciences). Conditions in the leaf cuvette were maintained as close as possible to the conditions in the growth cabinet, with VPD regulated by a portable dew point generator (LI-610, LI-COR Biosciences). Leaves were enclosed in the cuvette and instantaneous gas exchange was logged following stability in cuvette conditions (after ∼30 s). Following gas exchange measurements, the same leaf was then excised and immediately sampled for ABA quantification (see below).

### ABA purification and quantification

Samples harvested for ABA quantification were immediately weighed (±0.0001 g, MS204S, Mettler-Toledo) into 50 mL tubes, covered in ∼15 mL of cold (−20 °C) 80 % methanol in water (v v^−1^) with 250 mg L^−1^ (m v^−1^) of added butylated hydroxytoluene and transferred to −20 °C. Foliar ABA levels in these samples were extracted, purified and quantified by physicochemical methods using an added internal standard and UPLC-MS according to the methods of McAdam and Brodribb (2014).

### Gene isolation and phylogenetic analysis

Expressed sequence for *PsABA2* (GenBank accessions: GAMJ01000560, JI902419, JI907554, JR960889, JR962808) was identified by performing BLASTn searches using coding sequence for *MtABA2* (Medtr3g020670, v4.0, http://phytozome.jgi.doe.gov/) as a query against the *P. sativum* Transcriptome Shotgun Assembly sequences at GenBank (http://www.ncbi.nlm.nih.gov/), and used for primer design for *PsABA2*. Full-length coding sequence for *PsABA2* was isolated from genomic DNA in all *P. sativum* lines and *L. odoratus* with primers *PsABA2*-F 5′-TTGTGAGCCACCAACACTAC-3′ and *PsABA2*-R 5′-CACACACAATAAGGCACCTG-3′ (deposited at GenBank; accessions KT032072-KT032081). Amino acid sequence was predicted from exons identified through nucleotide alignment with expressed *PsABA2* sequence (GenBank accession KT032070), and predicted amino acid sequence was compared between lines. The putative mutation identified in *PsABA2* genomic DNA sequence from *wilty* lines was confirmed in *PsABA2* cDNA sequence from *wil* leaf material (GenBank accession KT032071).

To investigate the evolution of *ABA2* and the phylogenetic placement of *PsABA2*, amino acid sequences of SDRs from the SDR110C clade [sequence details in Moummou *et al*. (2012)] were assembled from 11 species spanning the diversity of the land plant phylogeny including *A. thaliana*, *Populus trichocarpa*, *Glycine max*, *Vitis vinifera*, *O. sativa*, *Z. mays*, *Sorghum bicolor*, *Selaginella moellendorffii* and *Physcomitrella patens* [sequences as per Moummou *et al*. (2012)], in addition to predicted protein sequences for SDR110C genes identified by reciprocal BLAST searches in *Picea abies* (http://congenie.org) and *Amborella trichopoda* (http://phytozome.jgi.doe.gov) for all known representatives). Amino acid sequences were aligned using ClustalX and distance and parsimony-based methods were used for phylogenetic analyses in PAUP version 4.0b10 (http://paup.csit.fsu.edu/).

### Statistical analysis

The effect of changing VPD on *g*_s_ and foliar ABA level of both wild-type and *wilty* mutant plants was tested by two-way analysis of variance (ANOVA). Pair-wise comparisons between the means of *g*_s_ and foliar ABA level for wild-type and mutant plants over the VPD transition were carried out using one-way ANOVAs followed by a Tukey's test. Analyses were performed using R Statistical Software.

## Results

### The *wilty* mutant carries a lesion in *PsABA2*

The phenotype of the *wilty* mutant implies that a gene involved in the ABA biosynthesis pathway may be compromised in this mutant. In previous studies, close synteny between the genomes of *P. sativum* and *Medicago* (Kaló *et al*. 2004) has enabled the use of publicly available *M. truncatula* sequence to select specific candidate genes for *P. sativum* mutant loci using a comparative mapping strategy (Hecht *et al*. 2005; Zhu *et al*. 2005). Strong linkage between *wilty* and the morphological markers *st* and *b* was detected in the earliest studies of the *wilty* mutant, indicating that the *WILTY* locus is located on *P. sativum* linkage group III (Marx 1976). A recent consensus map for *P. sativum*, which included tentative locations for known mutations, suggested that *st* may be located close to the marker gene *Pip1* (Bordat *et al*. 2011). We examined the corresponding region of the *M. truncatula* genome, searching for any genes known to be associated with ABA biosynthesis close to the *M. truncatula Pip1* orthologue (*MtPip1*; Medtr3g070210; v4.0, http://phytozome.jgi.doe.gov/) on *M. truncatula* chromosome 3. We identified a homologue of *ABA2* (*MtABA2*; Medtr3g020670) and further investigated the corresponding gene *PsABA2* as a candidate for *WILTY*.

**Figure 2.**
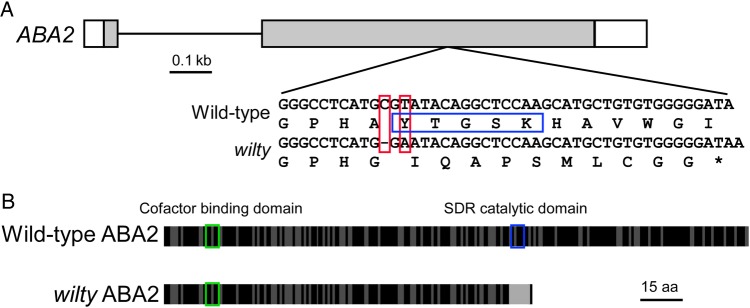
(A) The structure of the wild-type *P. sativum* (cultivar Torsdag) *ABA2* gene where grey boxes indicate exons, white boxes are untranslated regions and lines are introns. The nucleotide and corresponding amino acid change in the *wilty* mutant is shown below (red boxes indicating changes in sequence); the SDR-catalytic domain is highlighted by a blue box in the wild-type sequence. (B) The amino acid structure of the *ABA2* gene in wild-type lines of *P. sativum* and *L. odoratus* and the *ABA2* gene in *wilty* mutants (based on the alignment shown in **Supporting Information—Fig. S1**). Black bars represent amino acids that are conserved between wild-type *P. sativum* and *A. thaliana* ABA2 proteins; dark grey bars represent differences between these species; light grey bars represent amino acids unique to the *wilty* mutant. Key functional domains are shown in boxes including the cofactor binding domain (green) and the SDR-catalytic domain (blue) according to Joernvall *et al*. (1995).

Sequencing of *PsABA2* in eight diverse wild-type *P. sativum* lines, including a line containing the original *wilty* allele [L233 (Marx 1976)] and a near isogenic line derived by introgression of L233 with the cultivar Torsdag (*wil*), as well as the closely related legume *L. odoratus*, revealed a nucleotide change (CGT to GA) that causes a frameshift in codon 163 in *wilty* mutant plants relative to the wild-type *P. sativum* lines and *L. odoratus* (Fig. 2). This change, observed in both gDNA and cDNA, causes a premature stop after 11 missense amino acids, resulting in the loss of the highly conserved SDR-catalytic domain defined by the Tyr-XX-Ser-Lys motif (Fig. 2; **Supporting Information—Fig. S1**). In *A. thaliana*, mutation to this key domain results in the loss of ABA2 function in the *aba2-13* mutant (González-Guzmán *et al*. 2002). We predict that the mutation seen in the *P. sativum wilty* mutant results in loss of ABA2 function in a similar manner. The mutation in the *PsABA2* gene was carried by both the original L233 and the *wil* line, the result of introgression over five generations with the unrelated wild-type line Torsdag.

**Figure 3.**
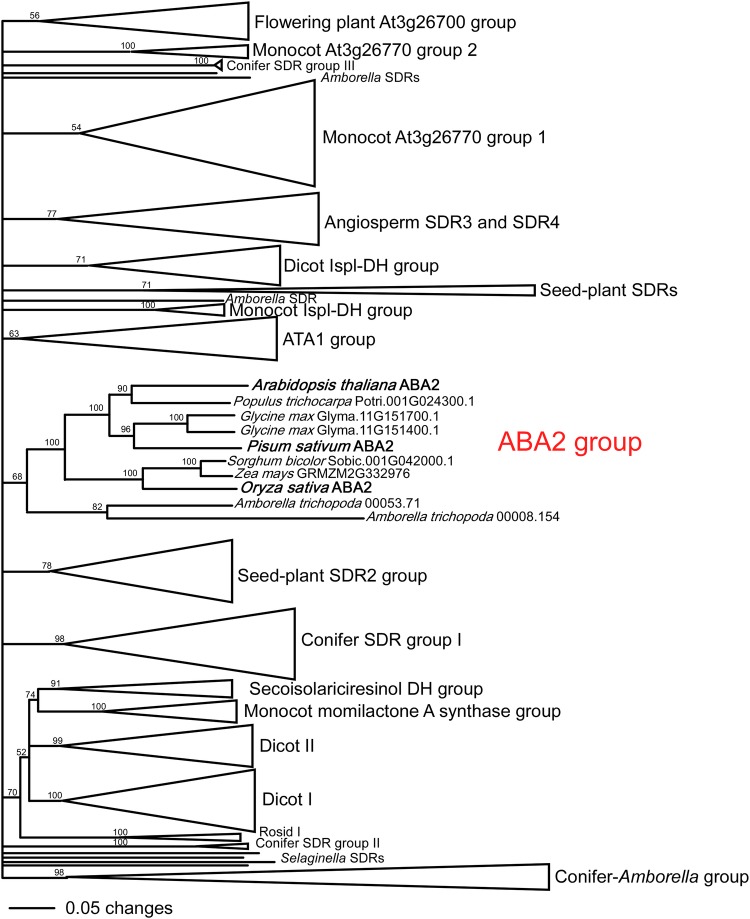
Phylogenetic tree of the SDR110C family of land plant SDRs, with individual representatives of the ABA2 clade shown including the characterized ABA-biosynthetic ABA2 enzymes from *A. thaliana* and *O. sativa* in bold. Bootstrap values from 1000 trees are shown next to each branch. Naming of angiosperm-specific clades follows that of Moummou *et al*. (2012). Full reference to the sequences compressed in each of the clusters is given in **Supporting Information—Fig. S2 and Table S1**.

Phylogenetic analysis showed that the *PsABA2* gene is embedded in a uniquely angiosperm clade of SDRs that includes the *ABA2* genes responsible for ABA biosynthesis in *A. thaliana* and *O. sativa* (Fig. 3). Intriguingly, this clade, dedicated to ABA biosynthesis, is only found in angiosperms. This clade is not represented in the conifer *P. abies*, lycophyte *S. moellendorfii* or moss *P. patens*, and the most basal genes in the *ABA2* clade are from the most basal angiosperm, *A. trichopoda* (Fig. 3). The *SDR* genes in non-flowering plants were no more closely related to the *ABA2* clade than to any of the other diverse clades of genes in the SDR110C family (Fig. 3).

### Dysfunctional stomatal response to changes in humidity in the *wilty* mutant

**Figure 4.**
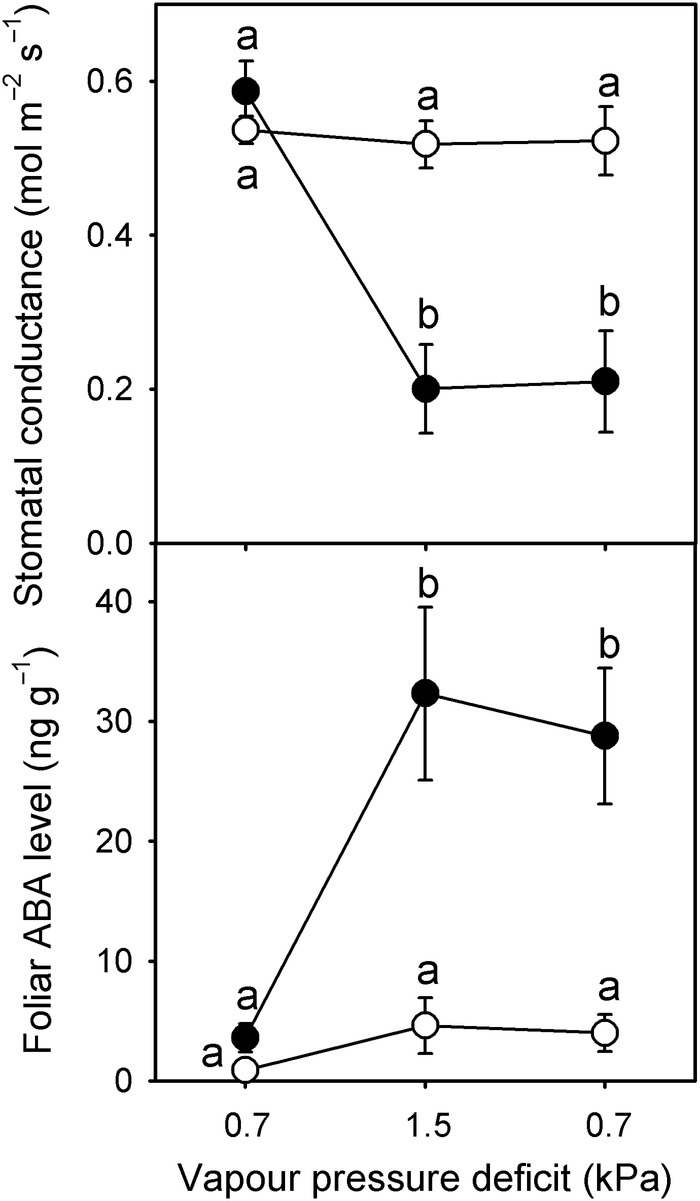
The mean response of stomatal conductance (*n* = 9 individuals, ±SE) and foliar ABA level (*n* = 9 individuals, ±SE) to a reversible sequence of VPD transitions from 0.7 to 1.5 kPa and returning to 0.7 kPa, with each transition lasting 20 min in wild-type (black circles) and *wilty* mutant (white circles) plants. Different letters denote significant difference between means (*P* < 0.05, One-way ANOVA followed by Tukey's test).

The stomata of *wilty* mutant plants do not have the characteristic rapid response to changes in VPD observed in wild-type *P. sativum* (Fig. 4; Table 1). In wild-type plants 20 min after a doubling in VPD (from 0.7 to 1.5 kPa), stomata closed significantly from an initial *g*_s_ of 0.587–0.2 mol m^−2^ s^−1^ (*F*_1, 4_ = 31.03, *P* = 0.0051; Fig. 4). This reduction in *g*_s_ in wild-type plants was accompanied by a significant 10-fold increase in foliar ABA levels (*F*_1, 16_ = 15.46, *P* = 0.0012; Fig. 4). Significantly higher foliar ABA levels in wild-type plants did not noticeably fall after 20 min on returning to a VPD of 0.7 kPa, resulting in pronounced hysteresis in the recovery of *g*_s_ (Fig. 4). In contrast to wild-type, the stomata of *wilty* plants did not close in response to a doubling in VPD and neither did foliar ABA levels significantly increase, remaining <5 ng g^−1^ fresh weight over the transition in VPD (Fig. 4; Table 2). Both *g*_s_ and foliar ABA levels in wild-type and *wilty* plants at the start of the experiment were not significantly affected by genotype (*g*_s_ *F*_1, 4_ = 0.27, *P* = 0.62; foliar ABA *F*_1, 16_ = 2.43, *P* = 0.14; Fig. 4).

**Table 1.** Summary of two-way ANOVA results for stomatal conductance measurements for wild-type and *wilty* plants during a reversible sequence of VPD transitions from 0.7 to 1.5 kPa and returning to 0.7 kPa, with each transition lasting 20 min.

	df	*F* value	*P* value
Genotype	1	46.71	1.81 × 10^−5^
VPD	2	22.05	9.59 × 10^−5^
Genotype × VPD	2	18.62	2.1 × 10^−4^
Residuals	12

**Table 2.** Summary of two-way ANOVA results for foliar ABA levels for wild-type and *wilty* plants during a reversible sequence of VPD transitions from 0.7 to 1.5 kPa and returning to 0.7 kPa, with each transition lasting 20 min.

	df	*F* value	*P* value
Genotype	1	29.90	1.61 × 10^−6^
VPD	2	9.06	4.60 × 10^−4^
Genotype × VPD	2	5.52	6.96 × 10^−4^
Residuals	48

## Discussion

Here we present evidence that the classical *wilty* mutant of *P. sativum* is due to a null mutation in the key ABA-biosynthetic gene, *ABA2*, which encodes a xanthoxin dehydrogenase (an SDR), responsible for the conversion of xanthoxin to abscisic aldehyde (Fig. 2). To summarize (i) both *wilty* lines in our study show severe deficiency in ABA biosynthesis, having very low levels in most tissues (Fig. 4) (de Bruijn *et al*. 1993*b*; Batge *et al*. 1999); (ii) the *ABA2* gene of *P. sativum* maps to the same chromosomal region as the *wilty* phenotype, at the base of linkage group III (Marx 1976); (iii) this *ABA2* gene of *P. sativum* is the only *ABA2* homologue in this species (Fig. 3); (iv) both of the *wilty* lines tested have a mutation in the key SDR-catalytic domain of the *ABA2* gene (Fig. 2), similar to the *aba2-13* mutant of *A. thaliana* and (v) the association between the *wilty* phenotype and this mutation in the *ABA2* gene has persisted through five generations of introgression with the wild-type line Torsdag. To date, the key SDR-mediated step in ABA biosynthesis has been represented only by mutants from a single species, *A. thaliana* (Fig. 1). Our finding that *wilty P. sativum* lines carry a severe mutation in *ABA2* strongly suggests that disruption to ABA2 function underlies the *wilty* mutant phenotype, resolving the unknown identity of this mutant. Taylor *et al*. (2000), suggested on the basis of Duckham *et al*. (1989), that the *wilty* mutant phenotype could be the result of a mutation in the *NCED* gene, responsible for the carotenoid cleavage step in ABA biosynthesis and the formation of xanthoxin (Fig. 1). However, the experiment of Duckham *et al*. (1989), which involved the feeding of abscisic aldehyde, the penultimate precursor in ABA biosynthesis, to *wilty* plants, could not rule out the possibility of *wilty* being a mutant for *ABA2*, as the formation of ABA following the feeding of abscisic aldehyde would be possible in mutants of both *ABA2* and *NCED* (Fig. 1). To rule out the unlikely possibility that another gene closely linked to *PsABA2* may be contributing to the *wilty* mutant phenotype, a future study could confirm restoration of phenotype in this mutant using wild-type *PsABA2*.

The most obvious feature of the *wilty* mutant phenotype is a rapid loss of turgor on exposure to conditions of increased evaporation (Wang *et al*. 1984). We show that this is because ABA levels increase in wild-type plants in response to low humidity, closing stomata, but not in the *wilty* mutant (Fig. 4). Mutants of *ABA2* in *A. thaliana* have a similar lack of stomatal closure under increased evaporative demand, with mutants of this gene identified as readily wilting (Léon-Kloosterziel *et al*. 1996). Mutants of the *ABA1*, *ABA2* and *ABA3* genes of *A. thaliana* display a varying degree of stomatal response to increased VPD (Assmann *et al*. 2000; Xie *et al*. 2006; Merilo *et al*. 2015), although these reports are not always consistent (compare the results of Bauer *et al*. 2013 with Merilo *et al*. 2015). However, as none of these studies measured ABA levels we cannot rule out the possibility that the *A. thaliana* biosynthetic mutants, all of which are known to have a degree of redundancy, had slight increases in foliar ABA levels at high VPD.

In addition to an obvious wilting phenotype, *aba2* mutants in *A. thaliana* also show reduced growth, which is presumably due to factors other than increased water stress, being observed even when plants are grown under very humid conditions (Cheng *et al*. 2002). In contrast, *wilty* mutant plants in *P. sativum* do not appear to have any obvious deficiency in shoot growth, but do exhibit reduced root growth (de Bruijn *et al*. 1993*a*). Whether this reduction in root growth in *wilty* plants is because ABA normally promotes root growth in wild-type plants, or is the result of increased water stress and lower water potentials in the mutant, is yet to be investigated.

Recently we have shown that stomatal responses to changes in humidity or VPD in angiosperms are driven by functionally significant changes in foliar ABA level; this is particularly evident in angiosperm herbs (McAdam and Brodribb 2015). Previously, some have argued that ABA responsible for the rapid (<1 h) responses of stomata to changes in water status does not have an origin in *de novo* synthesis but rather release from internal stores in the chloroplasts (Georgopoulou and Milborrow 2012) or via a single step that converts the catabolite ABA–glucose ester to ABA (Lee *et al*. 2006). Two findings in our study support an alternative view to these two hypotheses. The first is that our solvent-based extraction method for ABA does not discriminate between ABA that is found in the cytosol or bound in the chloroplasts, so any increase observed in the level of ABA in the leaf cannot be due to the redistribution of ABA between compartments of the leaf. The second is that the lack of a stomatal response to the step change in VPD in the *wilty* mutant plants was due to a lack of increase in the foliar ABA level. These observations suggest that the rapid (20 min), 10-fold increase in foliar ABA level in response to a doubling of VPD in *P. sativum* is likely the result of *de novo* synthesis. However, further work is required to investigate the importance of *de novo* biosynthesis in the rapid accumulation of ABA in response to a change in VPD.

An interesting observation emerging from the phylogenetic analysis of the SDR110C clade, which includes *ABA2* as well as related genes, is that a dedicated ABA-biosynthetic SDR (namely *ABA2*) evolved in the earliest angiosperms, including *A. trichopoda* (Fig. 3). This is surprising given that all clades of land plants are known to synthesize ABA (Johri 2008). Indeed, other studies investigating the evolution of ABA-biosynthetic genes have found that the genomes of the moss *P. patens* and the lycophyte *S. moellendorfii* also lack a specific *AAO* gene responsible for the final step in the biosynthetic pathway, the conversion of abscisic aldehyde to ABA (Hanada *et al*. 2011). There is, however, evidence that the conversion of xanthoxin to abscisic aldehyde can be catalyzed, to a very limited degree, by other SDRs in angiosperms. The null mutant of *Arabidopsis aba2-11*, for example, is still able to synthesize a small quantity of ABA (González-Guzmán *et al*. 2002). Indeed, unstressed *wilty* mutants grown under high humidity have similar foliar ABA levels compared with wild-type plants (Fig. 4). Also, in *wilty* mutants ABA levels begin to increase after 5 days of drought-stress, albeit to levels only one-third of those observed in wild-type plants (Wang *et al*. 1984). If the currently accepted biosynthetic pathway for ABA is conserved across land plants then the conversion of xanthoxin to abscisic aldehyde in basal clades of land plants is likely catalyzed by non-specific SDRs. This, as well as the lack of a specific *AAO* gene (Hanada *et al*. 2011), suggests that the final two steps in the ABA-biosynthetic pathway may be the rate-limiting steps for ABA biosynthesis in the basal lineages of land plants. This would stand in contrast to the hypothesized rate-limiting process for ABA biosynthesis in angiosperms, the carotenoid cleavage step leading to the formation of xanthoxin (Qin and Zeevaart 1999). Several observations indicating slow or delayed increases in foliar ABA level in these basal lineages support this possibility, including: (i) foliar ABA levels in ferns and lycophytes do not increase until plants approach a lethal water stress (McAdam and Brodribb 2013); (ii) foliar ABA levels in seedless vascular plants and conifers do not increase on exposure to increased VPD (McAdam and Brodribb 2015) and (iii) foliar ABA levels do not increase in the conifer *Metasequoia glyptostroboides* unless maintained for at least 6 h beyond turgor loss point (McAdam and Brodribb 2014), which is unlike ABA biosynthesis rates in angiosperms (Pierce and Raschke 1981). It could be argued that a single rate-limiting step in ABA biosynthesis, as occurs in angiosperms, would enable rapid and dynamic regulation of ABA biosynthesis. This would make it much easier for angiosperms to control a stomatal response to VPD through changes in the foliar ABA level. To date, however, there has been inadequate monitoring of ABA-biosynthetic gene expression during a VPD transition to confirm the single rate-limiting step hypothesis. It could be possible, in fact, that angiosperms dynamically alter the expression of multiple genes in the ABA-biosynthetic pathway in response to VPD (Thompson *et al*. 2007) but further work is required to investigate this scenario.

## Conclusions

Here we show that the classical ABA-biosynthetic mutant *wilty* in *P. sativum* contains a mutation in the ABA-biosynthetic gene *ABA2. wilty* mutant plants show no increase in foliar ABA levels during a whole-plant transition in VPD, and as a result do not close stomata. This lack of a stomatal response to changes in VPD strongly implicates ABA biosynthesis in the stomatal responses to changes in VPD in angiosperms. Phylogenetic analysis of the *ABA2* gene across land plants shows that the evolution of an ABA-biosynthetic-specific short-chain dehydrogenase (ABA2) evolved in the earliest angiosperms. This lack of specificity in the later-stage ABA-biosynthetic genes may explain why lycophytes, ferns and conifers, unlike angiosperms, do not show a rapid increase in foliar ABA levels on exposure to high VPD (McAdam and Brodribb 2015).

## Sources of Funding

Our work was funded by the Australian Research Council (DE140100946).

## Contributions by the Authors

S.A.M.M. designed and conducted physiological experiments, wrote the manuscript and prepared the figures; F.C.S. conducted molecular characterizations and wrote relevant sections of the manuscript; T.J.B. assisted in the design of the physiological experiments; J.J.R. assisted in experimental design including developing the protocol for hormone extraction and purification and supervising the development of isogenic lines; all authors provided critical review and revision of the manuscript.

## Conflict of Interest Statement

None declared.

## Supporting Information

The following additional information is available in the online version of this article–

**Figure S1.** Amino acid sequence alignment of the *ABA2* protein from *A. thaliana*, *L. odoratus* and eight diverse accessions and cultivars of *P. sativum* and the two lines carrying the *wilty* mutation.

**Figure S2.** Phylogenetic tree of the SDR110C family of land plant SDRs.

**Table S1.** Accession details for SDR110C family used to construct Figure 3 and **Supporting Information—Fig. S2**.

Additional Information
