# Supplementary material for: Molecular characterization of a mutation affecting abscisic acid biosynthesis and consequently stomatal responses to humidity in an agriculturally important species
Source: AoB Plants. 2015 Jul 27;7:plv091. doi: 10.1093/aobpla/plv091 (PMC4583606; doi:10.1093/aobpla/plv091)
Supplement: Additional Information [file supp_7_plv091_index.html]

Molecular characterization of a mutation affecting ABA biosynthesis and consequently stomatal responses to humidity in an agriculturally important species — Molecular characterization of a mutation affecting abscisic acid biosynthesis and consequently stomatal responses to humidity in an agriculturally important species — Additional Information 

# Molecular characterization of a mutation affecting abscisic acid biosynthesis and consequently stomatal responses to humidity in an agriculturally important species

## Additional Information

Additional Information

- Supplementary Figure 1 - doc file
- Supplementary Figure 2 - docx file
- Supplementary Table 1 - docx file
