# Supplementary material for: Molecular characterization of a mutation affecting abscisic acid biosynthesis and consequently stomatal responses to humidity in an agriculturally important species
Source: AoB Plants. 2015 Jul 27;7:plv091. doi: 10.1093/aobpla/plv091 (PMC4583606; doi:10.1093/aobpla/plv091)
Supplement: Additional Information [file supp_plv091_plv091supp_fig1.doc]

**Supporting Figure S1.** Amino acid sequence alignment of the *ABA2* protein from *Arabidopsis thaliana*, *Lathyrus odoratus* and eight diverse accessions and cultivars of *Pisum sativum* and the two lines carrying the *wilty* mutation. Shading in the sequence alignment indicates the degree of amino acid conservation (black = 100%, dark grey = 50%, light grey = 25%).

* 20 * 40 * 60 * 80 * 100
*Arabidopsis* ABA2 : MSTNTESSSYSSLPSQRLLGKVALITGGATGIGESIVRLFHKHGAKVCIVDLQDDLGGEVCKSLLRGESKETAFFIHGDVRVEDDISNAVDFAVKNFGTL : 100
*Lathyrus* LO5 : MSTTINA------PAQRLLGKVAVVTGGASGIGESIVRLFHSHGAKVCIADVQDYLGEKLCDSFC---DPENVYFVHCDVVVETDVSDAVYNTVGKFGTL : 91
*Pisum* cv. Virtus : MSTTINA------PAQRLLGKVAVVTGGASGIGASIVRLFHSHGAKVCIADVQDDLGQKLCDSFC---DPENVYFVHCDVAVETDVSDAVYNTVGKFGTL : 91
*Pisum* L107 : MSTTINA------PAQRLLGKVAVVTGGASGIGASIVRLFHSHGAKVCIADVQDDLGQKLCDSFC---DPENVYFVHCDVAVETDVSDAVYNTVGKFGTL : 91
*Pisum* cv. Cameor : MSTTINA------PAQRLLGKVAVVTGGASGIGASIVRLFHSHGAKVCIADVQDDLGQKLCDSFC---DPENVYFVHCDVAVETDVSDAVYNTVGKFGTL : 91
*Pisum* cv. Champagne : MSTTINA------PAQRLLGKVAVVTGGASGIGASIVRLFHSHGAKVCIADVQDDLGQKLCDSFC---DPENVYFVHCDVAVETDVSDAVYNTVGKFGTL : 91
*Pisum* Argenteum : MSTTINA------PAQRLLGKVAVVTGGASGIGASIVRLFHSHGAKVCIADVQDDLGQKLCDSFC---DPENVYFVHCDVAVETDVSDAVYNTVGKFGTL : 91
*Pisum* JI1771 : MSTTINA------PAQRLLGKVAVVTGGASGIGASIVRLFHSHGAKVCIADVQDDLGQKLCDSFC---DPENVYFVHCDVAVETDVSDAVYNTVGKFGTL : 91

*Pisum* JI281 : MSTTINA------PAQRLLGKVAVVTGGASGIGASIVRLFHSHGAKVCIADVQDDLGQKLCDSFC---DPENVYFVHCDVAVETDVSDAVYNTVGKFGTL : 91
*Pisum* cv. Kalisky : MSTTINA------PAQRLLGKVAVVTGGASGIGASIVRLFHSHGAKVCIADVQDDLGQKLCDSFC---DPENVYFVHCDVAVETDVSDAVYNTVGKFGTL : 91

***Pisum wilty wil*** : MSTTINA------PAQRLLGKVAVVTGGASGIGASIVRLFHSHGAKVCIADVQDDLGQKLCDSFC---DPENVYFVHCDVAVETDVSDAVYNTVGKFGTL : 91
***Pisum wilty* L233** : MSTTINA------PAQRLLGKVAVVTGGASGIGASIVRLFHSHGAKVCIADVQDDLGQKLCDSFC---DPENVYFVHCDVAVETDVSDAVYNTVGKFGTL : 91

 * 120 * 140 * 160 * 180 * 200
*Arabidopsis* ABA2 : DILINNAGLCGAPCPDIRNYSLSEFEMTFDVNVKGAFLSMKHAARVMIPEKKGSIVSLCSVGGVVGGVGPHSYVGSKHAVLGLTRSVAAELGQHGIRVNC : 200
*Lathyrus* LO5 : DIMVNNAGISGAPCPDIRNVDMSEFDKVFDINVKGVFHGMKHAAHVLIPKKSGSIISISSVASSLGGVGPHAYTGSKHAVWGITKNVAAELGNHGIRVNC : 191
*Pisum* cv. Virtus : DIMVNNAGISGAPCPDIRNVDMSEFDKIFDINVKGVFHGMKHAAHFLIPKKSGSIISISSVSSSLGGTGPHAYTGSKHAVWGITKNVAAELGNHGIRVNC : 191
*Pisum* L107 : DIMVNNAGISGAPCPDIRNVDMSEFDKIFDINVKGVFHGMKHAAHFLIPKKSGSIISISSVSSSLGGTGPHAYTGSKHAVWGITKNVAAELGNHGIRVNC : 191
*Pisum* cv. Cameor : DIMVNNAGISGAPCPDIRNVDMSEFDKIFDINVKGVFHGMKHAAHFLIPKKSGSIISISSVSSSLGGTGPHAYTGSKHAVWGITKNVAAELGNHGIRVNC : 191
*Pisum* cv. Champagne : DIMVNNAGISGAPCPDIRNVDMSEFDKIFDINVKGVFHGMKHAAHFLIPKKSGSIISISSVSSSLGGTGPHAYTGSKHAVWGITKNVAAELGNHGIRVNC : 191
*Pisum* Argenteum : DIMVNNAGISGAPCPDIRNVDMSEFDKIFDINVKGVFHGMKHAAHFLIPKKSGSIISISSVSSSLGGTGPHAYTGSKHAVWGITKNVAAELGNHGIRVNC : 191
*Pisum* JI1771 : DIMVNNAGISGAPCPDIRNVDMSEFDKIFDINVKGVFHGMKHAAHFLIPKKSGSIISISSVSSSLGGTGPHAYTGSKHAVWGITKNVAAELGNHGIRVNC : 191

*Pisum* JI281 : DIMVNNAGISGAPCPDIRNVDMSEFDKVFDINVKGVFHGMKHAAHFLIPKKSGSIISISSVSSSLGGTGPHAYTGSKHAVWGITKNVAAELGNHGIRVNC : 191
*Pisum* cv. Kalisky : DIMVNNAGISGAPCPDIRNVDMSEFDKVFDINVKGVFHGMKHAAHFLIPKKSGSIISISSVSSSLGGTGPHAYTGSKHAVWGITKNVAAELGNHGIRVNC : 191

***Pisum wilty wil*** : DIMVNNAGISGAPCPDIRNVDMSEFDKVFDINVKGVFHGMKHAAHFLIPKKSGSIISISSVSSSLGGTGPHGIQAPSMLCGG------------------ : 173
***Pisum wilty* L233** : DIMVNNAGISGAPCPDIRNVDMSEFDKVFDINVKGVFHGMKHAAHFLIPKKSGSIISISSVSSSLGGTGPHGIQAPSMLCGG------------------ : 173

 * 220 * 240 * 260 * 280 *
*Arabidopsis* ABA2 : VSPYAVATKLALAHLPEEERTEDAFVGFRNFAAANANLKGVELTVDDVANAVLFLASDDSRYISGDNLMIDGGFTCTNHSFKVFR---------- : 285
*Lathyrus* LO5 : VSPYCVATGLALAHLPEEERTEDAMEGFRSFVGKNANLQGVELTADDVANAVLFLASDDAKYISGENLMVDGGFTRTNHSLKVFR---------- : 276
*Pisum* cv. Virtus : VSPYGIATGLALAHLPEEERTEDVKAGFRSFVGKNANLQGVELTVDDVANAVLFLASDDAKYISGENLMVDGGFTRTNHSLKVFRG--------- : 277
*Pisum* L107 : VSPYGIATGLALAHLPEEERTEDVKAGFRSFVGKNANLQGVELTVDDVANAVLFLASDDAKYISGENLMVDGGFTRTNHSLKVFRG--------- : 277
*Pisum* cv. Cameor : VSPYGIATGLALAHLPEEERTEDVKAGFRSFVGKNANLQGVELTVDDVANAVLFLASDDAKYISGENLMVDGGFTRTNHSLKVFRG--------- : 277
*Pisum* cv. Champagne : VSPYGIATGLALAHLPEEERTEDVKAGFRSFVGKNANLQGVELTVDDVANAVLFLASDDAKYISGENLMVDGGFTRTNHSLKVFRG--------- : 277
*Pisum* Argenteum : VSPYGIATGLALAHLPEEERTEDVKAGFRSFVGKNANLQGVELTVDDVANAVLFLASDDAKYISGENLMVDGGFTRTNHSLKVFRG--------- : 277
*Pisum* JI1771 : VSPYGIATGLALAHLPEEERTEDVKAGFRSFVGKNANLQGVELTVDDVANAVLFLASDDAKYISGENLMVDGGFTRTNHSLKVFRG--------- : 277

*Pisum* JI281 : VSPYGIATGLALAHLPEEERTEDVKAGFRSFVGKNANLQGVELTVDDVANAVLFLASDDAKYISGENLMVDGGFTRTNHSLKVFRG--------- : 277

*Pisum* cv. Kalisky : VSPYGIATGLALAHLPEEERTEDVKAGFRSFVGKNANLQGVELTVDDVANAVLFLASDDAKYISGENLMVDGGFTRTNHSLKVFRG--------- : 277

***Pisum wilty wil*** : ----------------------------------------------------------------------------------------------- : -
***Pisum wilty* L233** : ----------------------------------------------------------------------------------------------- : -
