# Supplementary material for: Molecular characterization of a mutation affecting abscisic acid biosynthesis and consequently stomatal responses to humidity in an agriculturally important species
Source: AoB Plants. 2015 Jul 27;7:plv091. doi: 10.1093/aobpla/plv091 (PMC4583606; doi:10.1093/aobpla/plv091)

**Supporting Figure S2.** Phylogenetic tree of the SDR110C family of land plant SDRs, with individual representatives of the ABA2 clade shown including the characterised ABA biosynthetic ABA2 enzymes from *Arabidopsis thaliana* and *Oryza sativa* in bold. This tree was produced from an alignment of amino acid sequences using ClustalX from all SDR110C representatives from 11 land plant genomes spanning the diversity of land plants. Bootstrap values from 1000 trees are shown next to each branch.


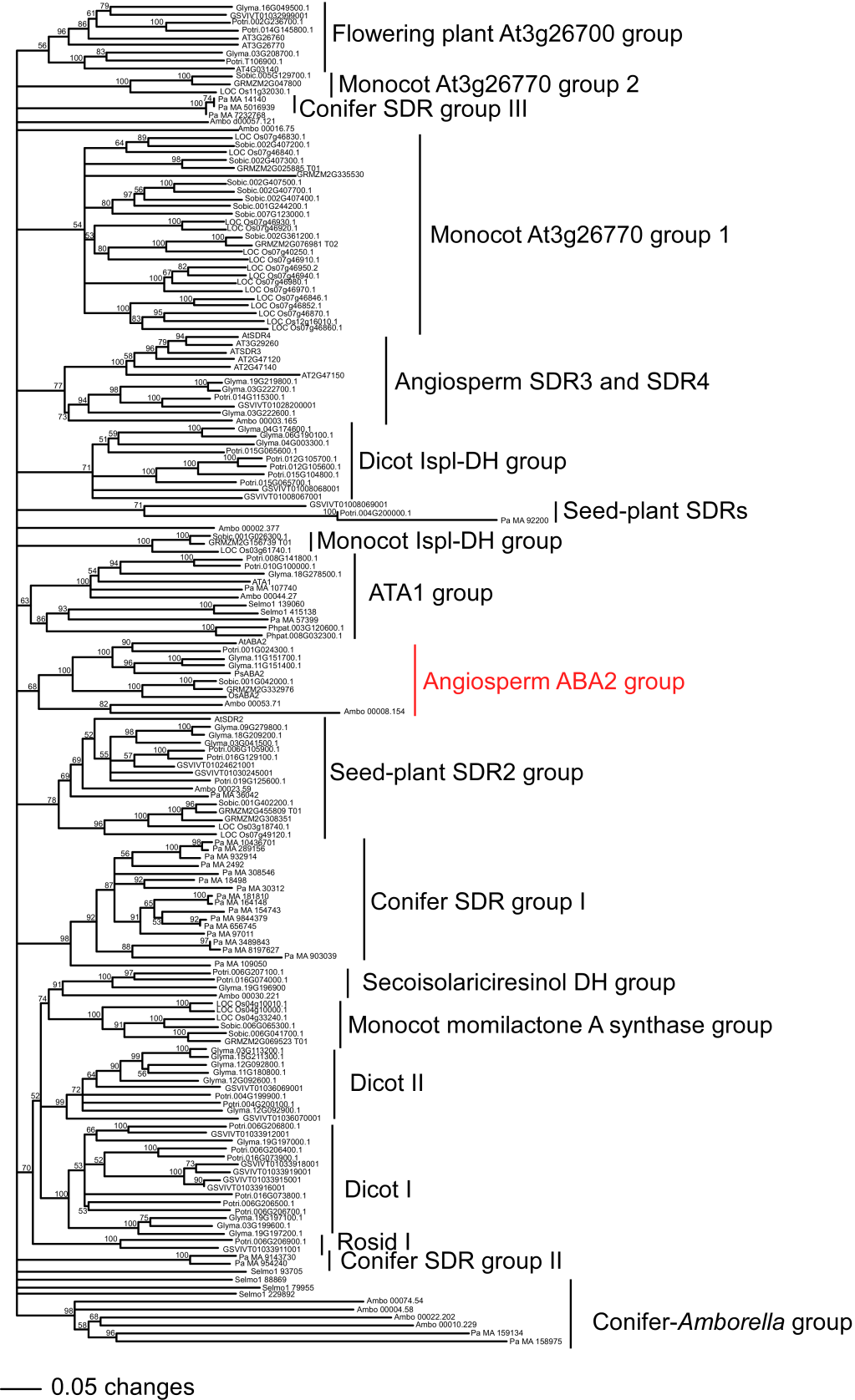

Supplement: Additional Information [file supp_plv091_plv091supp_fig2.docx]
