# Supplementary material for: Molecular characterization of a mutation affecting abscisic acid biosynthesis and consequently stomatal responses to humidity in an agriculturally important species
Source: AoB Plants. 2015 Jul 27;7:plv091. doi: 10.1093/aobpla/plv091 (PMC4583606; doi:10.1093/aobpla/plv091)
Supplement: Additional Information [file supp_plv091_plv091supp_table1.docx]

**Supporting Table S1.** Accession details and correspondence with details given in Moummou et al. 2012 for the Short-chain dehydrogenase/reductase 110C (SDR110C) family used to construct Figure 3 and Supporting Figure S2.

| **SDR family** | **Species** | **Locus** | **Name from Moummou et al. 2012** | | **NCBI** | |
| --- | --- | --- | --- | --- | --- | --- |
| SDR110C | Arabidopsis | AT3G51680 |  | | NP_190736 | |
| SDR110C | Arabidopsis | AT3G26770 |  | | NP_566798 | |
| SDR110C | Arabidopsis | AT3G26760 |  | | NP_189311.2 | |
| SDR110C | Arabidopsis | AT2G47130 |  | | NP_566798 | |
| SDR110C | Arabidopsis | AT4G03140 |  | | NP_567251.2 | |
| SDR110C | Arabidopsis | AT3G29260 |  | | NP_189571.1 | |
| SDR110C | Arabidopsis | AT3G29250 |  | | NP_189570.3 | |
| SDR110C | Arabidopsis | AT1G52340 |  | | NP_175644.1 | |
| SDR110C | Arabidopsis | AT3G42960 |  | | NP_189882.1 | |
| SDR110C | Arabidopsis | AT2G47120 |  | | NP_182234.1 | |
| SDR110C | Arabidopsis | AT2G47140 |  | | NP_566097.1 | |
| SDR110C | Arabidopsis | AT2G47150 |  | | NP_182237.1 | |
| SDR110C | Glycine | Glyma18g44060 | Glyma19g38370 | |  |  |
| SDR110C | Glycine | Glyma.03G113200.1 | Glyma03g26590 | |  |  |
| SDR110C | Glycine | Glyma.15G211300.1 | Glyma15g27630 | |  |  |
| SDR110C | Glycine | Glyma.12G092800.1 | Glyma12g09800 | |  |  |
| SDR110C | Glycine | Glyma.03G041500.1 | Glyma03g05070 | |  |  |
| SDR110C | Glycine | Glyma.03G208700.1 | Glyma03g36670 | |  |  |
| SDR110C | Glycine | Glyma.19G197000.1 | Glyma19g38380 | |  |  |
| SDR110C | Glycine | Glyma.19G197100.1 | Glyma19g38390 | |  |  |
| SDR110C | Glycine | Glyma.12G092600.1 | Glyma12g09780 | |  |  |
| SDR110C | Glycine | Glyma.03G199600.1 | Glyma03g35760 | |  |  |
| SDR110C | Glycine | Glyma.09G279800.1 | Glyma09g41620 | |  |  |
| SDR110C | Glycine | Glyma.18G209200.1 | Glyma18g44060 | |  |  |
| SDR110C | Glycine | Glyma.11G180800.1 | Glyma11g18570 | |  |  |
| SDR110C | Glycine | Glyma.04G003300.1 | Glyma04g00460 | |  |  |
| SDR110C | Glycine | Glyma.19G197200.1 | Glyma19g38400 | |  |  |
| SDR110C | Glycine | Glyma.19G219800.1 | Glyma19g40770 | |  |  |
| SDR110C | Glycine | Glyma.03G222700.1 | Glyma03g38160 | |  |  |
| SDR110C | Glycine | Glyma.11G151700.1 | Glyma11g21180 | |  |  |
| SDR110C | Glycine | Glyma.16G049500.1 | Glyma16g05400 | |  |  |
| SDR110C | Glycine | Glyma.04G174600.1 | Glyma04g34350 | |  |  |
| SDR110C | Glycine | Glyma.11G151400.1 | Glyma11g21160 | |  |  |
| SDR110C | Glycine | Glyma.12G092900.1 | Glyma12g09810 | |  |  |
| SDR110C | Glycine | Glyma.03G222600.1 | Glyma03g38150 | |  |  |
| SDR110C | Glycine | Glyma.18G278500.1 | Glyma18g51360 | |  |  |
| SDR110C | Glycine | Glyma.06G190100.1 | Glyma06g20220 | |  |  |
| SDR110C | Oryza | LOC_Os07g46840 |  |  |  |  |
| SDR110C | Oryza | LOC_Os03g61740 |  |  |  |  |
| SDR110C | Oryza | LOC_Os03g18740 |  |  |  |  |
| SDR110C | Oryza | LOC_Os04g10010 |  |  |  |  |
| SDR110C | Oryza | LOC_Os07g49120 |  |  |  |  |
| SDR110C | Oryza | LOC_Os04g10000 |  |  |  |  |
| SDR110C | Oryza | LOC_Os04g33240 |  |  |  |  |
| SDR110C | Oryza | LOC_Os07g46930 |  |  |  |  |
| SDR110C | Oryza | LOC_Os07g46830 |  |  |  |  |
| SDR110C | Oryza | LOC_Os11g32030 |  |  |  |  |
| SDR110C | Oryza | LOC_Os07g46920 |  |  |  |  |
| SDR110C | Oryza | LOC_Os03g59610 |  |  |  |  |
| **SDR family** | **Species** | **Locus** | **Name from Moummou et al. 2012** | | **NCBI** |  |
| SDR110C | Sorghum | Sobic.002G407300.1 | jgi\|Sorbi1\|5034810\|Sb02g042120 | |  |  |
| SDR110C | Sorghum | Sobic.002G407500. | jgi\|Sorbi1\|5034812\|Sb02g042140 | |  |  |
| SDR110C | Sorghum | Sobic.002G407700.1 | jgi\|Sorbi1\|5052203\|Sb02g042150 | |  |  |
| SDR110C | Sorghum | Sobic.002G407400.1 | jgi\|Sorbi1\|5034811\|Sb02g042130 | |  |  |
| SDR110C | Sorghum | Sobic.002G361200.1 | jgi\|Sorbi1\|5051952\|Sb02g038140 | |  |  |
| SDR110C | Sorghum | Sobic.005G129700.1 | jgi\|Sorbi1\|5040402\|Sb05g019130 | |  |  |
| SDR110C | Sorghum | Sobic.001G244200.1 | jgi\|Sorbi1\|5048846\|Sb01g021640 | |  |  |
| SDR110C | Vitis | GSVIVT01036069001 |  |  |  |  |
| SDR110C | Vitis | GSVIVT01030245001 |  |  |  |  |
| SDR110C | Vitis | GSVIVT01036070001 |  |  |  |  |
| SDR110C | Vitis | GSVIVT01032999001 |  |  |  |  |
| SDR110C | Vitis | GSVIVT01008068001 |  |  |  |  |
| SDR110C | Vitis | GSVIVT01033911001 |  |  |  |  |
| SDR110C | Vitis | GSVIVT01033918001 |  |  |  |  |
| SDR110C | Vitis | GSVIVT01033919001 |  |  |  |  |
| SDR110C | Vitis | GSVIVT01033915001 |  |  |  |  |
| SDR110C | Vitis | GSVIVT01033912001 |  |  |  |  |
| SDR110C | Vitis | GSVIVT01008067001 |  |  |  |  |
| SDR110C | Vitis | GSVIVT01033916001 |  |  |  |  |
| SDR110C | Vitis | GSVIVT01024621001 |  |  |  |  |
| SDR110C | Vitis | GSVIVT01028200001 |  |  |  |  |
| SDR110C | Vitis | GSVIVT01008069001 |  |  |  |  |
| SDR110C | Zea | GRMZM2G455809 |  |  |  |  |
| SDR110C | Zea | GRMZM2G332976 |  |  |  |  |
| SDR110C | Zea | GRMZM2G156739 |  |  |  |  |
| SDR110C | Zea | GRMZM2G069523 |  |  |  |  |
| SDR110C | Zea | GRMZM2G076981 |  |  |  |  |
| SDR110C | Zea | GRMZM2G047800 |  |  |  |  |
| SDR110C | Zea | GRMZM2G066840 |  |  |  |  |
| SDR110C | Zea | GRMZM2G308463 |  |  |  |  |
| SDR110C | Zea | GRMZM2G025885 |  |  |  |  |
| SDR110C | Zea | GRMZM2G308351 |  |  |  |  |
| SDR110C | Zea | GRMZM2G335530 |  |  |  |  |
| SDR110C | Zea | GRMZM2G308463 |  |  |  |  |
| SDR110C | Zea | GRMZM2G025885 |  |  |  |  |
| SDR110C | Zea | GRMZM2G308351 |  |  |  |  |
| SDR110C | Zea | GRMZM2G335530 |  |  |  |  |
| SDR110C | Amborella | AmTr_v1.0_scaffold00053.71 | |  |  |  |
| SDR110C | Amborella | AmTr_v1.0_scaffold00030.221 | |  |  |  |
| SDR110C | Amborella | AmTr_v1.0_scaffold00023.59 | |  |  |  |
| SDR110C | Amborella | AmTr_v1.0_scaffold00057.121 | |  |  |  |
| SDR110C | Amborella | AmTr_v1.0_scaffold00002.377 | |  |  |  |
| SDR110C | Amborella | AmTr_v1.0_scaffold00003.165 | |  |  |  |
| SDR110C | Amborella | AmTr_v1.0_scaffold00044.27 | |  |  |  |
| SDR110C | Amborella | AmTr_v1.0_scaffold00016.75 | |  |  |  |
| SDR110C | Amborella | AmTr_v1.0_scaffold00004.58 | |  |  |  |
| SDR110C | Amborella | AmTr_v1.0_scaffold00008.154 | |  |  |  |
| SDR110C | Amborella | AmTr_v1.0_scaffold00010.229 | |  |  |  |
| SDR110C | Amborella | AmTr_v1.0_scaffold00074.54 | |  |  |  |
| SDR110C | Amborella | AmTr_v1.0_scaffold00022.202 | |  |  |  |
| SDR110C | Picea | MA_2492 |  |  |  |  |
| SDR110C | Picea | MA_181810 |  |  |  |  |
| SDR110C | Picea | MA_97011 |  |  |  |  |

| **SDR family** | **Species** | **Locus** | **Name from Moummou et al. 2012** | **NCBI** |
| --- | --- | --- | --- | --- |
| SDR110C | Picea | MA_10436701 |  |  |
| SDR110C | Picea | MA_18498 |  |  |
| SDR110C | Picea | MA_164148 |  |  |
| SDR110C | Picea | MA_36042 |  |  |
| SDR110C | Picea | MA_9143730 |  |  |
| SDR110C | Picea | MA_954240 |  |  |
| SDR110C | Picea | MA_14140 |  |  |
| SDR110C | Picea | MA_903039 |  |  |
| SDR110C | Picea | MA_109050 |  |  |
| SDR110C | Picea | MA_308546 |  |  |
| SDR110C | Picea | MA_289156 |  |  |
| SDR110C | Picea | MA_107740 |  |  |
| SDR110C | Picea | MA_30312 |  |  |
| SDR110C | Picea | MA_57399 |  |  |
| SDR110C | Picea | MA_92200 |  |  |
| SDR110C | Picea | MA_932914 |  |  |
| SDR110C | Picea | MA_3489843 |  |  |
| SDR110C | Picea | MA_5016939 |  |  |
| SDR110C | Picea | MA_154743 |  |  |
| SDR110C | Picea | MA_8197627 |  |  |
| SDR110C | Picea | MA_656745 |  |  |
| SDR110C | Picea | MA_7232768 |  |  |
| SDR110C | Picea | MA_158975 |  |  |
| SDR110C | Picea | MA_159134 |  |  |
| SDR110C | Picea | MA_9844379 |  |  |
